# Activation of the ATM–Chk2 DNA damage response pathway by Newcastle disease virus enhances viral replication

**DOI:** 10.1186/s13567-025-01691-w

**Published:** 2026-01-30

**Authors:** Weiwen Yan, Chuanrong Dong, Xinxin Liu, Hongjin Li, JiaHuiZi Peng, Feng Jiang, Hongli Li, Tobias Stoeger, Abdul Wajid, Aleksandar Dodovski, Claro N. Mingala, Dmitry B. Andreychuk, Renfu Yin

**Affiliations:** 1 State Key Laboratory for Diagnosis and Treatment of Severe Zoonotic Infectious Diseases, Key Laboratory of Zoonosis Research, Ministry of Education, Department of Preventive Veterinary Medicine, College of Veterinary Medicine, Jilin University, Changchun, China; 2 College of Food Science and Engineering, Jilin University, Changchun, China; 3 College of Veterinary Medicine, Shanxi Agricultural University, Taiyuan, China; 4 Member of the German Center for Lung Research (DZL), Institute of Lung Health and Immunity (LHI), Comprehensive Pneumology Center (CPC), Helmholtz Zentrum München, Munich, Germany; 5 Department of Biotechnology, Balochistan University of Information Technology, Engineering and Management Sciences, Quetta, Pakistan; 6 Department for Avian Diseases, Faculty of Veterinary Medicine, Ss. Cyril and Methodius University in Skopje, Lazar Pop Trajkov 5-7, Skopje, Macedonia; 7 Livestock Biotechnology Center, Philippine Carabao Center, Science City of Muñoz, Nueva Ecija Philippines; 8 Reference Laboratory for Avian Viral Diseases, FGBI “Federal Centre for Animal Health” (FGBI “ARRIAH”), Vladimir, Russia

**Keywords:** Newcastle disease virus, DDR, replication, ATM–Chk2, cell cycle arrest

## Abstract

Newcastle disease (ND), caused by virulent strains of the Newcastle disease virus (NDV), is a highly contagious disease that poses significant economic burdens on the global poultry industry. The DNA damage response (DDR) is a critical cellular mechanism that detects and repairs genomic damage to maintain cellular integrity. While viral infections are known to modulate DDR pathways to either inhibit or enhance viral replication, the interaction between NDV and host DDR remains largely underexplored. Here, we demonstrate that NDV infection induces significant DNA damage in DF-1 cells and activates DDR signaling, primarily via the ataxia-telangiectasia mutated (ATM) kinase pathway, in a manner dependent on active viral replication. Pharmacological inhibition of ATM kinase, but not ataxia telangiectasia and Rad3-related (ATR) kinase, significantly suppresses NDV replication, alleviates virus-induced G1-phase cell cycle arrest, and modulates the host immune response. Moreover, short interfering RNA (siRNA)-mediated knockdown of *Chk2* markedly reduced viral *M* gene expression and progeny production, indicating that *Chk2* is required for efficient NDV replication. These findings suggest that NDV exploits the ATM–Chk2 DDR pathway to establish a replication-favorable environment. Our study provides new insights into NDV pathogenesis and highlights potential targets for antiviral interventions.

## Introduction

Poultry, the largest domestic animal stock globally, is a cornerstone of affordable, high-quality protein through eggs and meat, playing a vital role in global agriculture and economics. Small-scale poultry farming is particularly efficient, sustainable, and a reliable source of nutrition and income. However, Newcastle disease (ND), a highly contagious viral infection caused by *Avian orthoavulavirus* 1 (AOAV-1), commonly known as Newcastle disease virus (NDV), poses a significant threat to the poultry industry, inflicting substantial economic losses worldwide. NDV, a single-stranded, negative-sense, nonsegmented enveloped RNA virus of the subfamily *Avulavirinae* within the family *Paramyxoviridae* [1, 2], causes severe respiratory, neurological, and gastrointestinal symptoms in susceptible bird species. Despite widespread global vaccination programs, sporadic ND outbreaks persist [3–5], demonstrating the limitations of vaccines in fully preventing viral transmission and highlighting critical gaps in our understanding of NDV biology. A key factor contributing to ND’s persistence is the complex interplay between NDV and host cells, which drives viral replication and pathogenesis. Yet, the molecular mechanisms underlying these interactions remain poorly elucidated. A deeper understanding of NDV–host interactions at the molecular level is essential for identifying novel therapeutic targets and enhancing disease control strategies.

Upon viral infection, host cells initiate multiple defense mechanisms to counter invasion, including the DNA damage response (DDR). The DDR is a crucial cellular pathway that detects DNA damage, initiates repair, and maintains genomic stability [6]. Comprising sensors, transducers, and effectors, the DDR relies on key transducer kinases—ATM, ATR, and DNA-dependent protein kinase (DNA-PK)—which respond to specific DNA lesions. ATM and DNA-PK primarily respond to double-strand breaks (DSBs), whereas ATR is activated by single-strand DNA breaks (SSBs) and replication stress [7]. The DDR is vital for genomic integrity and can inhibit viral replication, but some viruses have evolved mechanisms to manipulate or suppress DDR pathways to enhance their replication [8]. While virus–DDR interactions are well studied in DNA viruses, such as human papilloma virus (HPV), which activates DDR to promote replication [9], or minute virus of mice (MVM), which inactivates the ATR pathway to accumulate damaged DNA [10], research on RNA viruses remains limited. For NDV, an important avian RNA virus, the specific interactions with host DDR pathways are largely unexplored. Understanding how NDV modulates DDR to facilitate replication could provide critical insights into its pathogenesis and guide the development of antiviral strategies.

This study investigates the interaction between NDV and host DDR pathways, focusing on their role in viral replication. We examined DDR activation in NDV-infected DF-1 cells, with particular emphasis on the ATM–Chk2 pathway. Our findings elucidate how NDV exploits host DDR mechanisms, offering novel perspectives on RNA virus pathogenesis and potential targets for improved antiviral strategies.

## Materials and methods

### Cell culture and NDV infection

DF-1 cells, obtained from the National Centre for Cell Science of China (NCCS), were cultured in Dulbecco’s modified Eagle medium (DMEM) supplemented with 10% fetal bovine serum (FBS; Gibco, USA) and 1% gentamicin-penicillin–streptomycin (Sigma, USA). Cells were maintained at 38 °C in a humidified incubator with 5% CO_2_. The virulent genotype VII NDV strain NA-1 (GenBank accession no. DQ659677) was propagated and maintained in our laboratory. For infection experiments, DF-1 cells were inoculated with NDV at a multiplicity of infection (MOI) of 0.2. Following a 2-h adsorption period, unattached viral particles were removed by washing three times with phosphate-buffered saline (PBS), and cells were cultured in complete medium. Infected cells were harvested at specified time points for downstream analyses.

### Preparation and validation of UV-inactivated NDV (UV-NDV)

Live NDV stocks (strain NA-1) were clarified by centrifugation (3000 × *g*, 10 min), aliquoted into 35-mm dishes at a fluid depth of approximately 2 mm (2.0 mL per dish), and placed on ice without lids. The virus was irradiated with UV-C light (254 nm) using a UV crosslinker at an intensity of ~1.2 mW cm^−2^ measured at the sample plane, to a cumulative dose of 1.2 J cm^−2^ (exposure time 1000 s; ~16 min 40 s) at a distance of 10 cm. Dishes were gently agitated every 3 min to ensure uniform exposure. Sham-irradiated controls were handled identically without UV. Immediately after irradiation, samples were aliquoted to avoid repeated freeze–thaw cycles, snap-frozen, and stored at −80 °C for no longer than 1 month. The absence of residual infectivity in UV-NDV preparations was verified using specific-pathogen-free (SPF) embryonated chicken eggs. Briefly, 9–10-day-old SPF embryos were inoculated via the allantoic cavity with 0.1 mL of UV-NDV suspension and incubated at 37 °C with daily candling. Sham-irradiated live NDV and phosphate-buffered saline (PBS) were used as positive and negative controls, respectively. At 72 h post-inoculation, allantoic fluids were harvested from both surviving and dead embryos and tested for hemagglutination (HA) activity using 1% chicken red blood cells. Complete inactivation was confirmed when no embryo mortality or HA activity was detected after two serial blind passages in SPF eggs.

### Alkaline comet assay

DNA damage was assessed using the alkaline comet assay, as previously described [11]. Briefly, mock- and NDV-infected DF-1 cells were harvested 24 h post-infection (hpi) and mixed with low-melting-point agarose. The mixture was spread onto slides precoated with normal agarose. Slides were subjected to electrophoresis at 25 V for 20 min in an alkaline buffer using an agarose gel electrophoresis system (Bio-Rad, USA). After staining with propidium iodide (PI), DNA damage was visualized under a fluorescence microscope. The extent of DNA damage was quantified using CASP software by analyzing randomly selected images from each group.

### Cytotoxicity assay

The cytotoxicity of ATM kinase inhibitor KU55933 and ATR kinase inhibitor VE-821 (MCE, USA) in DF-1 cells was assessed using the CCK-8 Cell Activity Detection Kit (Mei5 Biotechnology, China), according to the manufacturer’s protocol. Approximately 20,000 cells were seeded in 96-well plates 24 h prior to the experiment. At 80% confluence, cells were treated with varying concentrations of inhibitors for 12 h, with dimethyl sulfoxide (DMSO) as the vehicle control. Subsequently, 10 µL of CCK-8 reagent was added to each well, and cells were incubated for 1–4 h. Absorbance was measured at 450 nm, and cell viability was calculated as: cell viability (%) = [(As − Ab)/(Ac − Ab)] × 100%, where As is the absorbance of the experimental well, Ac is the absorbance of the control well, and Ab is the absorbance of the blank well.

### Reverse transcription-quantitative PCR (RT-qPCR)

Total RNA was extracted from cells using TRIzol reagent (Sigma, USA) and reverse-transcribed into complementary DNA (cDNA) using a reverse transcription kit. Gene expression levels were quantified by real-time quantitative PCR (qPCR) on an ABI StepOne PCR system (Applied Biosystems, USA) using the Quick Start Universal SYBR Green Master kit (Roche, Switzerland). Relative gene expression was calculated using the 2^−∆∆Ct^ method, normalized to the housekeeping gene β-actin, as previously described [12].

### siRNA-mediated silencing of *Chk2*

Given that *Chk2* was prioritized from six NDV-upregulated ATM–DDR candidates owing to its pivotal role as a direct ATM effector that links DNA damage to checkpoint control [7], it was subsequently selected for siRNA-mediated silencing in DF-1 cells. Cells were seeded in six-well plates at 70–80% confluence and transfected with Lipofectamine 3000 (Thermo Fisher Scientific, USA) according to the manufacturer’s instructions. Two Chk2-specific siRNA sequences (sense strand: 5′-GCUGGAGUUUAAGAGUUAUTT-3′ and 5′-GCGGUAAAGAUAAUCAAUATT-3′) were used, with a scrambled siRNA (sense strand: 5′-UUCUCCGAACGUGUCACGUTT-3′) as a negative control. All siRNAs were designed and synthesized by GenePharma, China. At 24 h post-transfection, cells were infected with NDV at an MOI of 0.2. Supernatants were collected 24 hpi for 50% tissue culture infectious dose (TCID_50_) assays, and total RNA was extracted from cells for gene expression analyses.

### Cell cycle analysis

Cell cycle analysis was conducted as previously described [13]. Mock- and NDV-infected DF-1 cells were harvested, washed three times with precooled PBS, and fixed in 70% ethanol at −20 °C. Fixed cells were stained with a propidium iodide (PI) solution containing RNase A and analyzed by flow cytometry (fluorescence-activated cell sorting (FACS)). The cell cycle distribution in G1/G0, S, and G2/M phases was determined on the basis of DNA content.

### Statistical analysis

Statistical analyses were performed using GraphPad Prism version 10.1.2 (GraphPad Software, USA). Data from three independent experiments are presented as mean ± standard deviation (SD). Differences between groups were assessed using an unpaired two-tailed Student’s *t*-test, with significance denoted as *P < 0.05, **P < 0.01, and ***P < 0.001.

## Results

### NDV infection, but not inactivated NDV, induces a DNA damage response via the ATM DDR pathway

**Figure 1:**
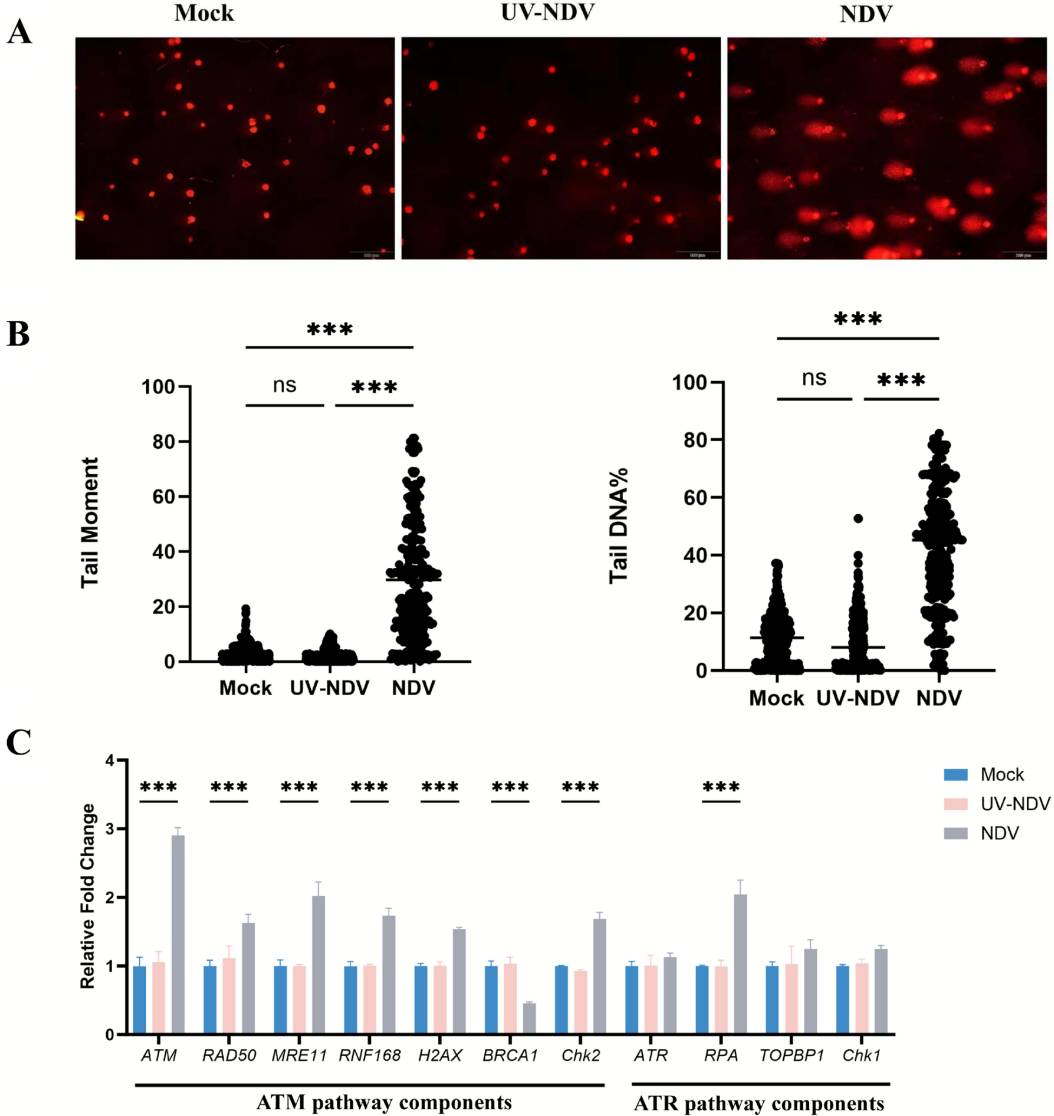
**NDV infection induces DNA damage in DF-1 cells primarily through the ATM DDR pathway.** Mock-, UV-inactivated NDV (UV-NDV)-, and NDV-infected DF-1 cells were harvested at 24 hpi, and DNA damage was assessed using the alkaline comet assay. **A** Representative comet assay images illustrating DNA damage in Mock, UV-NDV, and NDV-infected cells. **B** Cluster plot analysis of tail moments in mock-, UV-NDV-, and NDV-infected cells, reflecting the extent of DNA damage. **C** Relative mRNA levels of DDR-related genes in mock-, UV-NDV, and NDV-infected DF-1 cells at 24 hpi were quantified by RT-qPCR, normalized to β-actin. Genes include ATM pathway components (*ATM*, *RAD50*, *MRE11*, *RNF168*, *H2AX*, *BRCA1*, and *Chk2*) and ATR pathway components (*ATR*, *RPA*, *Chk1*, and *TOPBP1*). Data from three independent experiments are shown as mean ± standard deviation (SD). Statistical significance is denoted as **P < 0.01; ***P < 0.001.

Viruses, as nucleic acid-based obligate intracellular microorganisms, exploit host cell processes, including the evolutionarily conserved DDR signaling pathway, to facilitate proliferation and evade antiviral defenses [8, 14]. To determine whether NDV infection induces host DNA damage, we performed an alkaline comet assay to evaluate DNA integrity in NDV-infected DF-1 cells at 24 hpi. As shown in Figure 1A and B, mock-infected cells displayed minimal DNA migration and damage, whereas NDV-infected cells exhibited significantly increased DNA migration and extensive DNA damage, evidenced by elevated tail moment and tail DNA percentage. Notably, UV-inactivated NDV (UV-NDV) did not induce DNA damage, suggesting that the DDR is driven by replication stress associated with active viral infection.

To further investigate NDV-induced DDR pathway activation, we analyzed transcript levels of key DDR-related genes in NDV-infected DF-1 cells at 24 hpi. These genes included ATM-pathway components (*ATM*, *RAD50*, *MRE11*, *RNF168*, *H2AX*, *BRCA1*, and *Chk2*) and ATR-pathway components (*ATR*, *RPA*, *Chk1*, and *TOPBP1*). Consistent with the comet assay results, 72.73% (8/11) of DDR-related genes—*ATM*, *RAD50*, *MRE11*, *RNF168*, *H2AX*, *BRCA1*, *CHK2*, and *RPA*—were significantly altered in NDV-infected cells compared with mock-infected controls, indicating DDR activation (Figure 1C). Six of these genes (*ATM*, *RAD50*, *MRE11*, *RNF168*, *H2AX*, and *CHK2*) were upregulated and associated with the ATM DDR pathway, while *RPA*, linked to the ATR pathway, was also upregulated. However, *BRCA1*, a critical ATM-pathway gene involved in homologous recombination repair (HRR), was significantly downregulated in NDV-infected cells (Figure 1C). In contrast, UV-NDV-infected cells showed no significant changes in DDR gene expression compared with mock-infected controls (Figure 1C). These findings collectively demonstrate that active NDV infection, but not inactivated NDV, induces a host DDR predominantly via the ATM pathway, likely conferring a proliferative advantage to the viral life cycle.

### The ATM DDR pathway facilitates efficient NDV proliferation

**Figure 2:**
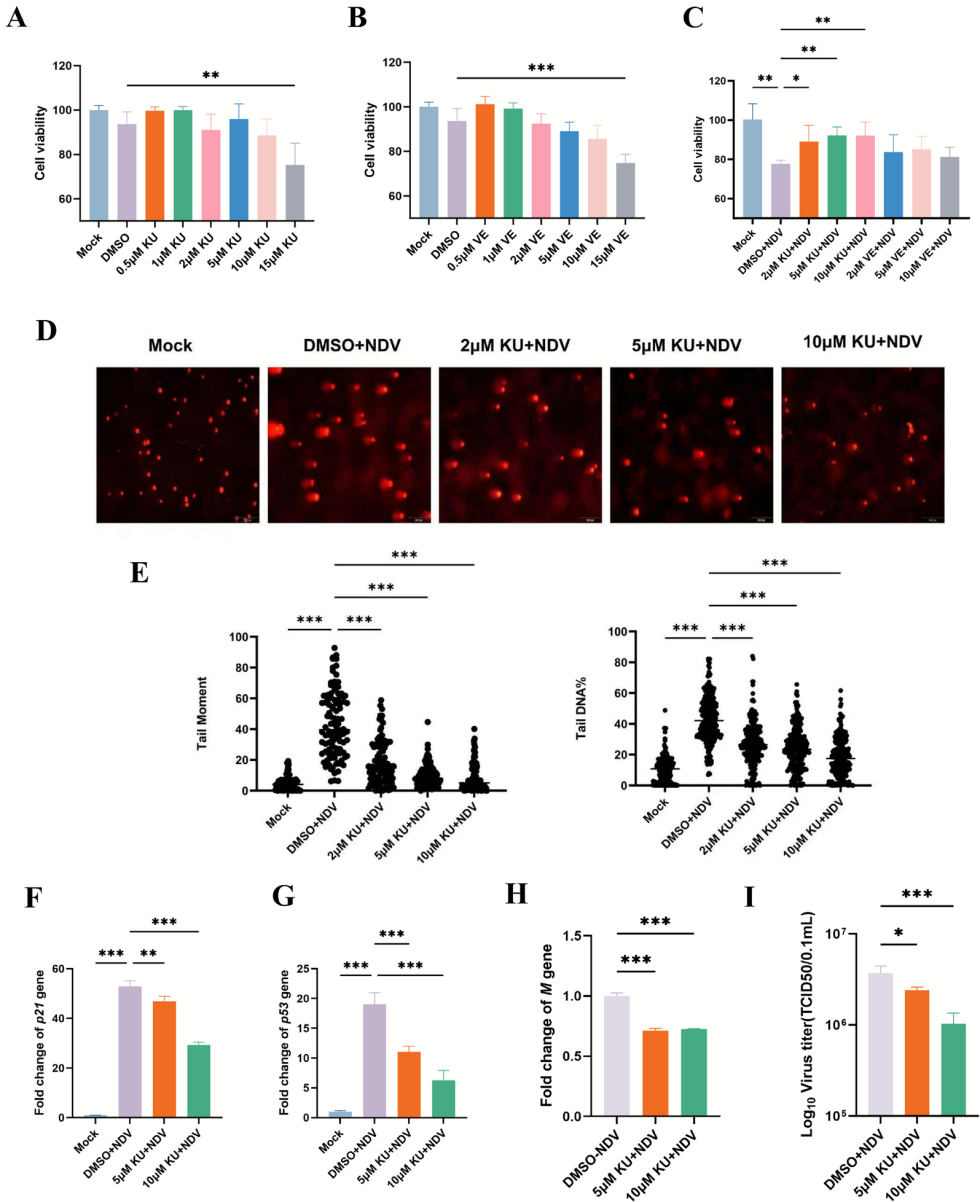
**ATM kinase inhibitor suppresses NDV proliferation**. **A**, **B** DF-1 cells were pretreated with ATM kinase inhibitor KU55933 or the ATR kinase inhibitor VE-821 at varying concentrations for 2 h, and cell viability was assessed using the CCK-8 assay. **C** Cell viability in KU55933-pretreated NDV-infected cells or VE-821-pretreated NDV-infected cells was evaluated using the CCK-8 assay. **D**, **E** DNA damage in mock-infected cells, DMSO-pretreated NDV-infected cells, and KU55933-pretreated NDV-infected cells was assessed via alkaline comet assay. **D** Representative comet images and **E** cluster plot analysis of tail moments illustrate DNA damage extent. **F**, **G** Total RNA was extracted from mock-infected, DMSO-pretreated NDV-infected, and KU55933-pretreated NDV-infected cells, and *P21* and *P53* expression levels were quantified by RT-qPCR, shown as fold changes. **H** NDV *M* gene expression was analyzed by RT-qPCR. **I** Viral titers in cell supernatants are presented as bar diagrams. Data from three independent experiments are shown as mean ± SD. Statistical analysis used an unpaired two-tailed Student’s *t*-test, with *P < 0.05, **P < 0.01, and ***P < 0.001.

To investigate the role of the DDR pathway in NDV infection, we conducted a CCK8 assay to evaluate cell viability and the anti-NDV effects of the ATM kinase inhibitor KU55933 and the ATR kinase inhibitor VE-821 in DF-1 cells. Cell viability remained above 90% at drug concentrations up to 10 μM for 2 h, comparable to mock controls, for both inhibitors (Figures 2A and B). However, pretreatment with KU55933 (2 to 10 μM) significantly reduced NDV’s impact on cell activity at 24 hpi compared with VE-821 or the vehicle control (DMSO) (Figure 2C). Furthermore, NDV-induced DDR activation, including downstream genes *p21* and *p53*, was significantly reduced in KU55933-pretreated cells in a dose-dependent manner compared with DMSO controls (Figures 2D–G). Similarly, NDV *M* gene expression and viral titers were significantly lower in KU55933-pretreated cells, with viral titers exhibiting a dose-dependent reduction (Figures 2H, I). These findings demonstrate that ATM kinase inhibition suppresses NDV replication, highlighting the critical role of the ATM DDR pathway in facilitating efficient NDV proliferation.

### Chk2 is crucial for NDV proliferation

**Figure 3:**
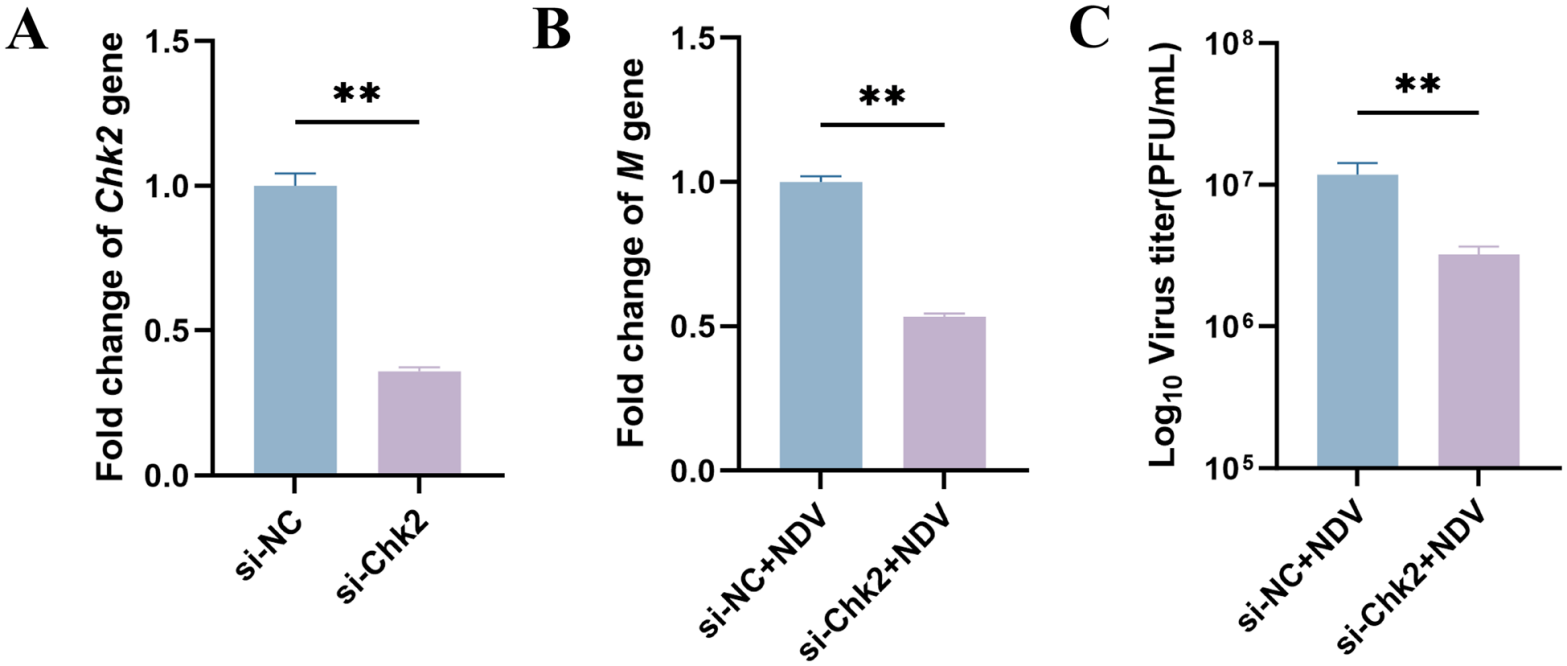
**Chk2 is essential for efficient NDV proliferation**. DF-1 cells were transfected with 100 pmol of scramble siRNA (si-NC) or Chk2-specific siRNA (si-Chk2) to evaluate Chk2’s role in NDV-induced DDR and virus infection. **A** *Chk2* mRNA levels were quantified by RT-qPCR, with si-NC-transfected cells as the control. **B**, **C** At 24 h post-transfection, cells were infected with NDV and harvested at 24 hpi. **B** Fold changes in NDV *M* gene expression were measured by RT-qPCR. **C** Viral titers in cell supernatants were measured. Data from three independent experiments are presented as mean ± SD. Statistical analysis used an unpaired two-tailed Student’s *t*-test, with **P < 0.01.

Chk2, a key downstream effector of the ATM DDR signaling pathway, is activated in response to DNA damage. To investigate its role in NDV-induced DDR and viral infection, *Chk2* expression was knocked down in DF-1 cells using 100 pmol of small interfering RNA (siRNA). At 24 h post-transfection (hpt), RT-qPCR analysis revealed a 64% reduction in Chk2 expression compared with the scrambled siRNA control (Figure 3A). Subsequently, siRNA-transfected cells were infected with NDV (MOI 0.2), and supernatants and cells were collected at 24 hpi for TCID_50_ and RT-qPCR analysis, respectively. Chk2-silenced cells exhibited a 67% reduction in viral progeny production (Figure 3B) and a 53% decrease in NDV M gene expression compared with controls (Figure 3C). These findings demonstrate that Chk2 is a critical host factor for NDV proliferation.

### NDV infection induces G1-phase cell cycle arrest

**Figure 4:**
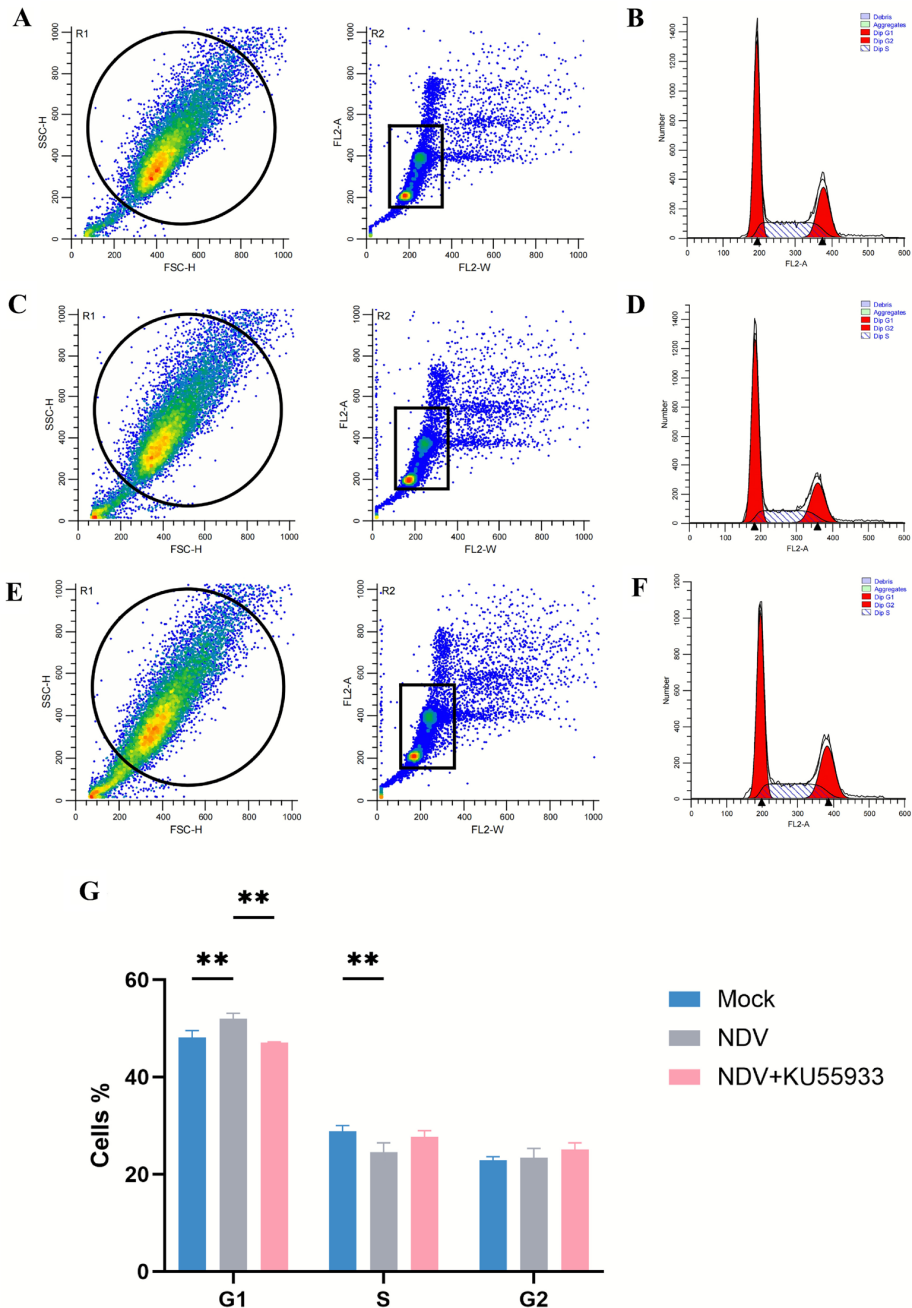
**NDV infection induces G1-phase cell cycle arrest**. **A**, **C**, **E** Gating strategy for cell-cycle analysis. DF-1 cells collected at 24 hpi were stained with propidium iodide (PI) and analyzed by flow cytometry. R1 (FSC-H versus SSC-H) defines the primary cell population; R2 (FL2-A versus FL2-W) selects singlets. Panels show representative gating from the mock- (**A**), NDV-infected (**C**), and KU55933-pretreated NDV-infected groups (**E**). The same gates were applied to all samples. Debris and doublets were excluded by the R1 ∩ R2 gates. **B**, **D**, **F** Representative PI DNA-content histograms (FL2-A) from the corresponding groups: mock (**B**), NDV (**D**), and KU55933-pretreated NDV (**F**). Deconvolution curves illustrate the contributions of G1, S, and G2/M phases. **G** Mock-infected, NDV-infected, and KU55933-pretreated NDV-infected DF-1 cells were collected at 24 hpi. Cell cycle distribution was analyzed by propidium iodide staining and flow cytometry. Bar graphs represent the percentage of cells in G1, S, and G2 phase. Data from three independent experiments are presented as mean ± SD. Statistical analysis used an unpaired two-tailed Student’s *t*-test, with *P < 0.05 and **P < 0.01.

Many viruses manipulate the host cell cycle to optimize replication. Given that the DDR pathway genes, including downstream regulators *p53* and *p21*, were significantly altered in NDV-infected cells (Figures 1C, 2F, G), we investigate NDV’s impact on the cell cycle. Using propidium iodide (PI) staining, we measured DNA content in mock- and NDV-infected cells at 24 hpi. NDV infection significantly increased the proportion of cells in the G1 phase while reducing those in the S phase, indicating G1-phase cell cycle arrest (Figure 4). Pretreatment with the ATM inhibitor KU55933 significantly alleviated this arrest, suggesting that NDV-induced DDR, mediated by the ATM pathway, contributes to G1-phase cell cycle arrest, likely facilitating viral replication.

### ATM signaling modulates host innate immunity and NDV replication

**Figure 5:**
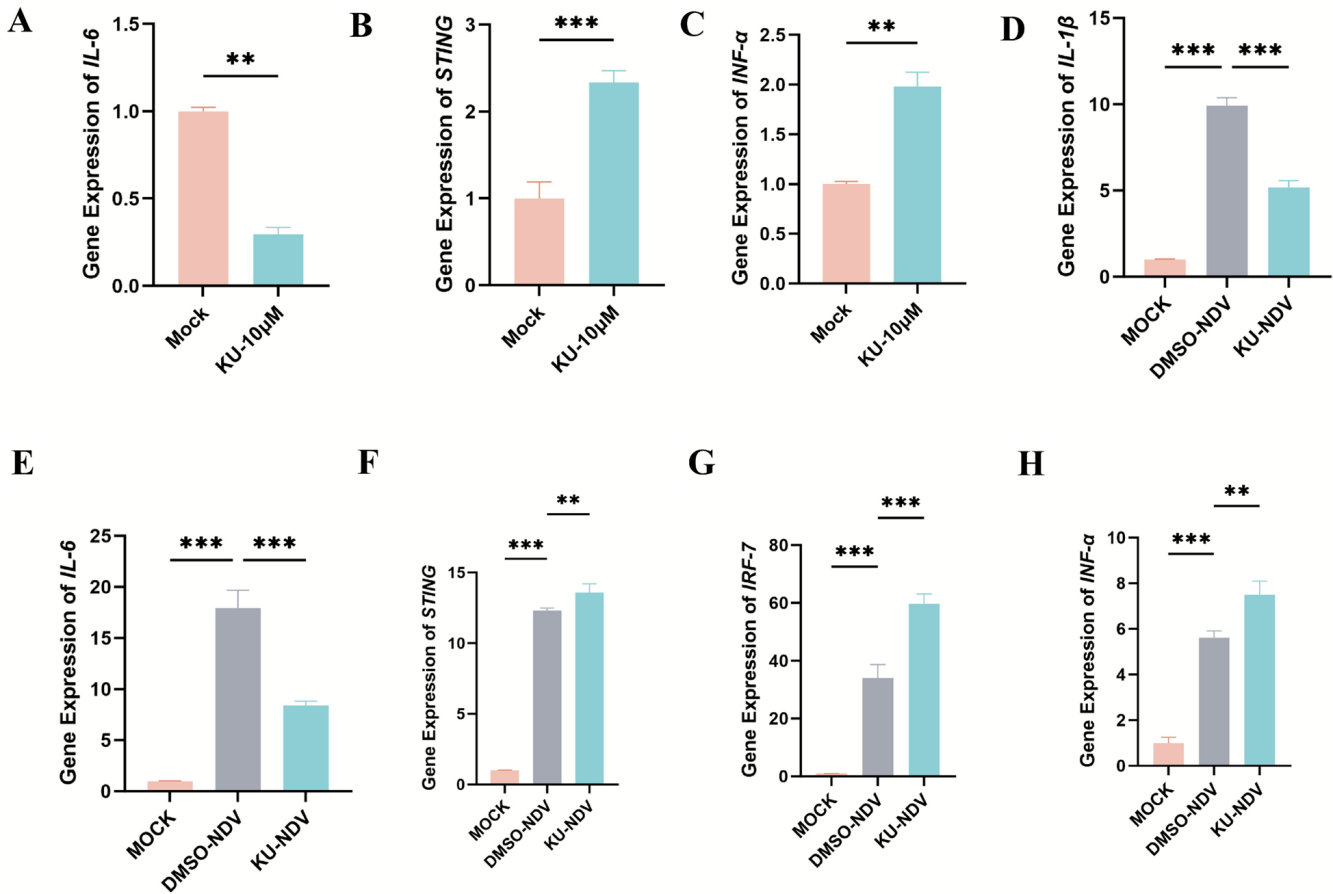
**ATM inhibition modulates host innate immune genes expression**. **A**–**C** DF-1 cells were treated with 10 μM ATM inhibitor KU55933, and relative mRNA levels of interferon signaling and pro-inflammatory cytokine genes were quantified by RT-qPCR. **D**–**H** Relative mRNA levels of interferon signaling and pro-inflammatory cytokine genes were measured in mock-infected cells, NDV-infected cells pretreated with DMSO, and KU55933-pretreated NDV-infected cells by RT-qPCR. Data from three independent experiments are presented as mean ± SD. Statistical analysis used an unpaired two-tailed Student’s *t*-test with **P < 0.01 and ***P < 0.001.

Previous experiments demonstrated that the ATM inhibitor KU55933 significantly reduced NDV *M* gene expression and viral titers, highlighting the role of the ATM pathway in NDV replication (Figures 2H, I). To elucidate how ATM regulates host antiviral responses, we employed RT-qPCR to assess expression changes in interferon pathway genes and pro-inflammatory cytokines in KU55933-treated DF-1 cells. Compared with untreated controls, ATM-inhibited cells exhibited significantly reduced interleukin-6 (*IL-6*) expression, alongside marked upregulation of stimulator of interferon genes (*STING*) and interferon-alpha (*IFN-α*) (Figures 5A–C), suggesting a link between ATM signaling and interferon responses.

To further evaluate the role of ATM in NDV-induced innate immunity, we compared gene expression profiles in NDV-infected versus uninfected DF-1 cells. NDV infection robustly increased expression of pro-inflammatory cytokines *IL-1* and *IL-6*, as well as interferon pathway genes *STING*, *IRF-7*, and *IFN-α*. In ATM inhibitor-treated infected cells, *IL-6* and *IL-1* expression was significantly suppressed, while *STING*, *IRF-7*, and *IFN-α* levels were further elevated (Figures 5D–H). These results indicate that ATM inhibition not only restricts NDV replication but also modulates the host immune response by suppressing pro-inflammatory cytokine production and enhancing interferon signaling. This mechanism likely contributes to the observed reduction in NDV replication, reinforcing the critical role of ATM-mediated DDR in regulating host antiviral immunity.

## Discussion

**Figure 6:**
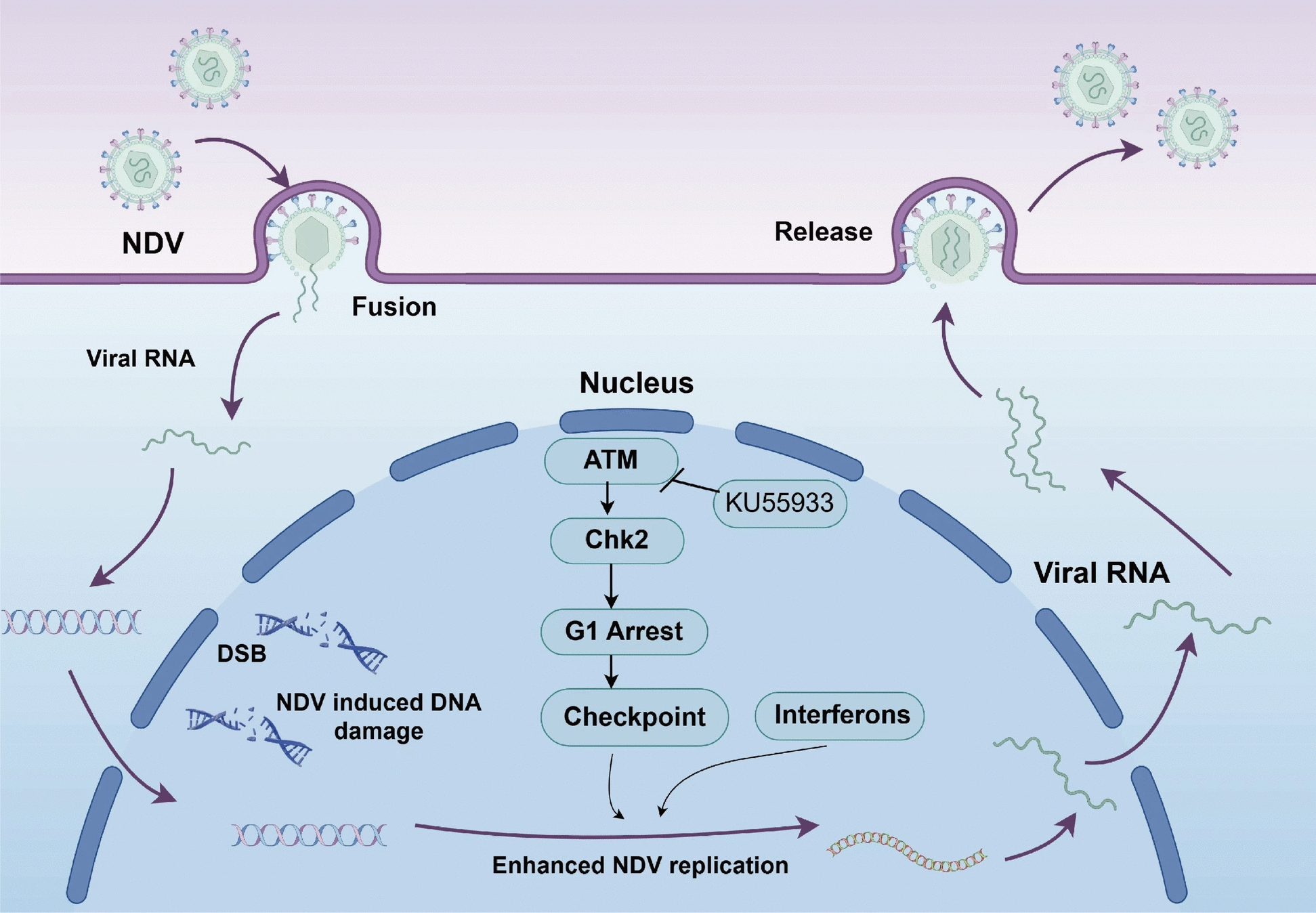
**Model of NDV modulation of the ATM–Chk2 DDR pathway for efficient proliferation**. This study demonstrates that NDV infection triggers a significant DDR, activating the ATM–Chk2 signaling pathway. Inhibition with KU55933 significantly reduces NDV replication. NDV infection also causes G1-phase cell cycle arrest, likely facilitating viral replication. Schematic created using FigDraw.

Viruses, as obligate intracellular parasites, manipulate host cell machinery to replicate their genomes and produce proteins, often by hijacking the host’s DDR pathways [13, 14]. While virus–DDR interactions have been extensively studied in DNA viruses [15, 16], research on RNA viruses, including NDV, remains limited. This study suggests that NDV infection triggers a robust host DDR, primarily through the ATM–Chk2 DDR pathway, which promotes viral replication by modulating cell cycle progression and innate immune responses (Figure 6). Consistent with previous findings showing that NDV activates the ATM–Chk2 axis to enhance viral replication and syncytium formation in human tumor cells, our observations in avian cells suggest that this mechanism is conserved across different host systems [17]. In contrast to DNA viruses—which often manipulate the DDR to facilitate genome synthesis or circumvent checkpoint control (e.g., adenoviral inactivation of the MRN complex; the context-dependent suppression and co-option of DDR factors by herpes simple virus (HSV)-1; the restraint of Chk2 to prevent G2/M arrest by Epstein–Barr virus (EBV))—reports of RNA-virus-induced DNA damage remain comparatively limited. However, emerging evidence shows that severe acute respiratory syndrome coronavirus 2 (SARS-CoV-2) and influenza viruses can elicit DSB signaling, while Seneca Valley virus (SVV) engages ATM/ATR/DNA-PK and disrupts repair-focus formation to favor replication [18–23]. These findings offer valuable insights into the pathogenesis of NDV and its interplay with host cellular processes, though further research is required to fully elucidate the underlying mechanisms.

RNA viruses cause numerous widespread and often severe diseases. Although their replication typically occurs in the cytoplasm, several RNA viruses, such as SARS-CoV-2 and influenza, induce DNA damage and activate DDR pathways [18, 24, 25]. Our results demonstrate that NDV, a significant avian RNA virus, triggers substantial DDR, whereas UV-inactivated NDV does not, suggesting that active viral replication is essential for this response. Previous studies have demonstrated that NDV strains of varying virulence elicit distinct immune responses and replication dynamics [26–28]. Given the interplay between DDR and innate immunity, DDR activation likely varies among NDV strains. Our findings indicate that the virulent genotype VII NDV strain (NA-1) robustly activates the ATM pathway, enhancing replication (Figures 1C, 2H, I). Whether this is a universal feature of NDV or specific to virulent strains remains unclear. Future investigations comparing NDV strains across the virulence spectrum, combined with functional analyses of DDR components, will clarify whether DDR modulation is a conserved mechanism or strain-specific.

Notably, *Breast Cancer Gene 1* (*BRCA1*), a key ATM-pathway gene involved in homologous recombination repair (HRR), was downregulated in NDV-infected cells (Figure 1C). This suppression may delay DNA repair checkpoints, creating a replication-favorable environment for NDV [29, 30]. Conversely, replication protein A (*RPA*), an ATR-pathway factor critical for replication, recombination, and repair, was upregulated (Figure 1C), a phenomenon associated with enhanced replication in viruses such as minute virus of mice and vaccinia virus [31, 32]. These findings suggest that NDV selectively modulates DDR components to support its replication, warranting further investigation into the roles of *BRCA1* and *RPA* in NDV pathogenesis.

The DDR consists of three major pathways—ATM, ATR, and DNA-PK [33, 34]—and viruses often target specific pathways to optimize replication. For instance, SARS-CoV-2 and Marek’s disease virus (MDV) suppress the ATR–Chk1 pathway [11, 35], while Epstein–Barr virus (EBV) modulates ATM–Chk2 [20]. Similarly, Chikungunya virus (CHIKV) activates both ATM and ATR pathways, with its nsP2 protein interacting with Chk2 and Chk1 [13]. Our study demonstrates that the ATM inhibitor KU55933, but not the ATR inhibitor VE-821, significantly reduces NDV replication, consistent with findings in hepatitis C virus [36]. This indicates that the ATM kinase is critical for NDV propagation, while the ATR pathway is dispensable. Although differences in cell viability between ATM and ATR inhibitor treatments were modest, consistent trends across experiments indicate biological significance. The pronounced effect of ATM inhibition likely reflects NDV-induced DNA damage, which preferentially activates the ATM pathway, as supported by viral titer and cytokine expression data.

Certain viruses exploit DDR effectors to enhance replication. For instance, the Vif protein of human immunodeficiency virus (HIV)-1 blocks ATM activation [37], while Pseudorabies virus (PRV) and Enterovirus 71 (EV71) regulate γ-H2AX levels [38, 39]. Our study found that Chk2 silencing significantly reduced NDV *M* gene expression and viral progeny production, confirming Chk2’s role as a critical host factor for NDV proliferation. NDV-induced cellular stress may activate ATM–Chk2, prolonging cell cycle arrest to extend the replication window. Mechanistically, Chk2 functions as a pro-replicative checkpoint hub. By promoting Cdc25A degradation and activating the p53–p21 axis, it enforces and sustains G1/S arrest, thereby restricting host DNA synthesis while maintaining the high translational capacity characteristic of G1-phase cells [40, 41]. Once DNA damage signaling is initiated, sustained Chk2 activity can uphold G1 arrest even with limited upstream input, consistent with our proposed cascade—ATM → Chk2 → G1 maintenance → enhanced viral yield—and with the minimal impact observed following ATR inhibition. This model is further supported by evidence from other viral systems: inhibition of ATM/Chk2 reduces HSV-1 production, while CHIKV engages Chk2/Chk1 via nsP2, with Chk2 silencing markedly decreasing progeny yields [13, 42]. Collectively, these results suggest that targeting Chk2 could limit NDV replication, though whether Chk2 directly or indirectly interacts with NDV proteins requires further exploration.

Many viruses, including Japanese encephalitis virus (JEV), influenza, SARS-CoV-2, and murine hepatitis virus (MHV), induce G1-phase cell arrest via ATM–Chk2 [43–46], while others, such as Bocavirus, Epstein–Barr virus (EBV), and HIV, arrest at G2 [47, 48]. Our study shows that NDV infection significantly increases G1-phase cells in DF-1 cells, indicating G1-phase arrest (Figure 4). The G1-phase is characterized by high transcriptional activity of RNA polymerase II (Pol II) and translational efficiency [49, 50], which NDV may exploit to enhance protein synthesis and replication. By arresting the cell cycle in G1, NDV secures resources and delays apoptosis. Although the increase in G1-phase cells was modest, its correlation with Chk2 modulation and viral titers suggests that cell cycle regulation influences NDV replication. Future time-course and single-cell analyses will further validate this mechanism.

DDR pathways also interact with the host antiviral immune system. ATM inhibition can enhance STING-dependent type I IFN response [51], while viruses such as herpes simplex virus type 1 (HSV-1) exploit DDR–immune cross-talk to suppress defenses [52]. NDV suppresses IFN signaling to facilitate replication [53, 54]. Our study shows that ATM inhibitor significantly alters interferon and cytokine expression, reducing IL-6 and IL-1 while upregulating IFN-α, IRF-7, and STING in NDV-infected DF-1 cells (Figure 5D–H). This suggests that ATM inhibition enhances innate antiviral responses by relieving IFN suppression, restricting NDV replication. NDV likely exploits ATM signaling to dampen immunity, similar to chikungunya virus (CHIKV) [13]. These findings highlight the role of ATM in shaping NDV’s replication environment.

A limitation of this study is the reliance on mRNA expression to assess DDR component activation (ATM, ATR, and Chk2), which may not fully reflect kinase activity. The lack of DF-1-cell-compatible antibodies for detecting phosphorylated ATM, ATR, or Chk2 (Thr68) hindered protein-level analysis. Future studies utilizing single-cell phosphoproteomics or optimized antibodies will address these constraints and clarify DDR dynamics during NDV infection.

In conclusion, NDV infection activates the ATM–Chk2 pathway, resulting in DNA double-strand breaks, G1-phase cell cycle arrest, and altered innate immune responses to promote replication. ATM inhibition with KU55933, but not ATR inhibition, suppresses NDV replication while upregulating interferon expression, indicating that NDV exploits ATM–Chk2 to coordinate host cell cycle and immune modulation (Figure 6). These findings offer new insights into NDV’s manipulation of host DDR pathways and a theoretical basis for antiviral strategies targeting NDV and other RNA viruses. The specific role of Chk2 in NDV infection remains unclear, and proteomic studies are needed to uncover direct interactions. Further exploration of ATM–Chk2 regulators during NDV infection may reveal novel antiviral targets, advancing our understanding of RNA virus–host interactions.

## Data Availability

All data supporting the findings of this study are openly available in Ref. [55] and via Figshare at 10.6084/m9.figshare.29453261. The data are shared under a CC-BY license and are freely accessible without restriction.
